# A cluster analysis of high-performance female team players’ perceived motivational climate: Implications on perceived motor competence and autonomous behaviour

**DOI:** 10.1371/journal.pone.0278572

**Published:** 2022-12-06

**Authors:** J. Arturo Abraldes, Luis Conte Marín, David Manzano-Sánchez, Manuel Gómez-López, Bernardino J. Sánchez-Alcaraz

**Affiliations:** Department of Physical Activity and Sport, University of Murcia, Murcia, Spain; Rio de Janeiro State University: Universidade do Estado do Rio de Janeiro, BRAZIL

## Abstract

High performance sport for females is an area which is gaining more and more relevance today, but which hasn’t received the same research interest as sport for males. The aim of the present study was to analyse the motivational climate perceived by high performance female athletes and the implications on perceived motor competence and autonomous behaviour and check the differences according category, sport experience and training hours in performance and master climate. The participants were 615 female athletes who practice top level team sports, with ages comprised of 16 to 38 (M = 22,10; SD = 4,91). The sample consisted of two different categories: junior (n = 242) and senior (n = 373). These players participated in different team sports, specifically football, handball, basketball and volleyball, training between 6 and 24 hours a week (M = 8,34; DT = 4,33). The variables measured were: perceived motivational climate in sport, autonomous behaviour and perceived motor competence. A cluster analysis was carried out using, as a variable, the perceived motivational climate, and showing the existence of two profiles, one related to ego and the other to task. The multivariate analysis showed that the profile orientated towards the task had significant differences compared to the autonomous behaviour (M = 4.66 vs M = 3.41). At the same time an analysis was carried out looking at different social demographic variables, revealing how there were differences in the sports experience (those participants with more than ten years’ experience were more orientated towards ego, compared to those with less than ten years’ experience) and the category (junior athletes were more orientated towards the task, compared to senior athletes). It was concluded that a greater orientation towards the task can lead to an improvement in the perception of motor competence, with the youngest and least experienced athletes being the most prominent in this category.

## Introduction

Sport has always been present, throughout history. Nowadays everyone is encouraged to participate, without discriminating against anyone [1]. Certain groups, like female athletes who practice team sports, have been studied little, despite being an interesting area. Besides, in sport in general, success and failure come from a combination of physical, technical, tactical and psychological qualities, the last of which are far-reaching in sports achievements [2–4]. So, in the last few years, research around motivation has been framed within the perspective of diverse motivational theory, with at least 32 theories [5] which try to explain why people are or are not motivated to carry out their activity [6].

The Theory of Achievement Goals [7, 8], indicates to us those various situational factors that exist (such as motivational climate), and which determine the achievement context in which the individual finds him or herself, together with their personal characteristics, which will influence their final implication, as these factors will orientate the participant towards either the performance or the task. A figure of authority, such as the trainer, is essential for both the development of self-motivation [6] and for psychological well-being [9], as well as the satisfaction of the athletes [10].

In this way, the perception of a climate orientated towards the task, favoured by the trainer, can predict the competence as well as the support towards perceived autonomy [11], both of which are two of the basic psychological needs.

The theory of basic psychological needs is worth pointing out, as it considers that the human being needs to satisfy three basic psychological needs, which are essential for optimum function, specifically, the need for independence, social relationships, and competence. The perception of competence can be the one which is least damaged by a motivational climate orientated towards self. It is for this reason that, in competitive situations, it can result that the players are not capable of perceiving this climate in a match, when the pressure is higher [12]. On the other hand, Bortoli et al. [13], point out that perceived competence is the strongest predictor of a more positive psychosocial state in athletes, as well as a more self-determined motivation [14] and subjective vitality [15]. While support for independence means that the trainer is able to put him/her self into the players’ perspective in order to detect their needs and offer them the opportunity to choose [9].

This need is the essential element to satisfy the basic psychological needs [16–19] and will help to obtain a more self-determined motivation [15, 16]. In this way Alesi et al. [20] show how the satisfaction of both needs are what are most related to a climate of orientation of self and commitment to sport. In the same way, Claver et al. [21] in an intervention with female volleyball players observed how, from the three basic psychological needs, independence and perceived competence were what improved most significantly.

Focusing on the perceived motivational climate and motivation in team sports, it is clear that the figure of the trainer is fundamental in improving this [22–25]. There are studies which point out differences between the motive to practice and the orientation of motivational climate, with a bigger orientation towards the ego of men [24–28] and some women [29].

Other studies, in women, reflect that the majority of players, despite belonging to the high-performance level, are usually more involved in the task, and have a more intrinsic than extrinsic motivation, as much in football [30] as in basketball [31].

According to Aguirre-Loaiza et al. [32], the years of sports experience or the competitive level don’t appear to influence motivational orientation. However, it has been proven that the passing of time increases the demands on the sports tasks, and makes the athletes develop certain characteristics in order to face up to these challenges. Furthermore, the higher level of weekly sports activity is related to the higher amount of motivational climate implicated to task [33], in the same way as when the competitive level increases [34].

Due to the existence of a smaller number of studies in the female sports context, the purpose of this study was to analyse the motivational climate perceived by high performance female athletes. and the implications on perceived motor competence and autonomous behaviour. Finally, we contrast the differences between the variables, depending on the category of play, the sports experience and the time spent training per week in perceived motivational climate.

## Materials and methods

### Study design and participants

A transversal and quantitative study was carried out and informed consents (confidential data treatment, participation in the study) were requested from the high-performance athletes. Participants were 615 high-performance female athletes, aged between 16 and 38 years (M = 22.10; SD = 4.91). The sample was recruited from two different categories: junior (n = 242) and senior (n = 373). These players participated in different high-performance team sports, such us football, handball, basketball or volleyball, training between 6 and 24 hours per week (M = 8.34; SD = 4.33). Participant characteristics are included in Table 1.

**Table 1:** Participant characteristics.

	AGE		
	N	M	SD
Total sample	*615*	*22*.*10*	*4*.*91*
Category			
Junior	242	17.18	2.41
Senior	373	24.09	1.21

### Procedure

In order to collect the data, the organizing federations and the participating national teams were informed, and the necessary written informed consent was obtained. Players completed the three questionnaires (Perceived Motivational Climate in Sport Questionnaire, Autonomy-Supportive Coaching Questionnaire and Physical Self-concept Scale) voluntarily and anonymously in a classroom, before a team practice. Previously, one researcher had had a meeting with the different coaches to teach them how to carry out the questionnaires. The coach of each team was asked to leave the room while the questionnaires were being completed, which took about 20 minutes, and none of players reported any problems completing them. Insofar as ethical rules are concerned, the study previously received the approval of the Ethics Committee of the University of Murcia (ID: 1494/2017). All participants were treated in agreement with the ethical guidelines regarding consent, confidentiality and anonymity of the answers.

### Instruments

#### Motivational climate

The Spanish version of the Perceived Motivational Climate in Sport Questionnaire (PMCSQ-2) [35], developed by Balaguer et al. [36] was used. The inventory includes 29 items grouped in two dimensions measuring the ego-involving climate or performance climate (14 items) with three subscales, unequal recognition, punishment for mistakes and intrateam rivalry and competition; and the task-involving climate or mastery climate dimension (15 items) with another three subscales, emphasis on effort and improvement, perceived important role and cooperative learning. Each item was headed with the phrase ‘‘In my training group or team…” Answers were collected on a Likert type scale ranging from strongly disagree (1) to strongly agree (5). Internal consistency analysis yielded satisfactory results for both mastery (α = .86) and performance dimensions (α = .85).

#### Autonomous behavior

The factor, praise for autonomous behavior, from the Spanish version [37] from Conroy and Coatsworth’s [38], Autonomy-Supportive Coaching Questionnaire (ASCQ) was used. This factor comprises four items that measure how players evaluate praise for their autonomy. The questionnaire began with the phrase "In my training sessions…”. The responses were limited to a Likert scale from 1 (strongly disagree) to 7 (strongly agree). The internal consistency obtained .81.

#### Perceived competence

The sports competence factor perceived through the Physical Self-concept Scale (PSQ) was used [39]. This scale is composed of 30 items distributed in five sections (perceived competence, physical appearance, physical condition, physical strength and self-belief). This comes from the original Physical Self-Perception Profile (PSPP) [40–42]. The factor used consisted of six items which ended the sentence “When I practice sport I ……” Answers were collected on a Likert type scale ranging from strongly disagree (1) to strongly agree (4). Perceived sports competence obtained a Cronbach alfa value of .83.

### Data analysis

Descriptive and correlation analysis of all variables included in the study was conducted. A hierarchical cluster analysis using Ward’s method was performed to create several groups based on the coach-created motivational climate [43]. Squared Euclidean distance between observation was computed as the dissimilarity measure. The NbClust R package was used to determine the optimal number of clusters of the dataset [44]. The stability of the clusters was determined by randomly dividing the study sample into two halves to perform a hierarchical (Ward’s method) analysis on one half, an iterative (K-means) analysis on the other half and a final K-mean cluster on the total sample [43]. The Pearson’s correlation coefficient (r) was used to measure associations between scale items. Each profile’s features were examined through a multivariate analysis of variance (MANOVA) of the complete sample. Chi-Square test was used to examine age, sport experience and training hours differences between the obtained clusters. The strength of associations was studied by computing the adjusted standardized residuals (z), considering values >1.96 as little, >2.58 as weak and >3.29 as strong associations [45]. Cramer’s V correlation coefficient was calculated as a measure of the effect size (ES), considering 0.10 = small effect, 0.30 = medium effect, and 0.50 = large effect [46]. The level of significance was set at p < .05. Calculations were made using IBM SPSS Statistics for Macintosh (Version 25.0. Armonk, NY: IBM Corp.) and the psych [47] and NbClust [44] packages for R (version 3.6.1).

## Results

### Descriptive statistic and correlations

Descriptive statistics, Cronbach’s alpha values for the subscales and bivariate correlations for all the study variables are presented (see Table 2). The data reveal higher scoring of mastery climate compared with performance climate (M = 4.05, 2.85, respectively), as well as medium levels of perceived motor competence (M = 4.13) and autonomous behavior (M = 4.08). Regarding motivational climate, results showed higher values in cooperative learning, emphasis on effort and improvement and perceived important role, and lower values of unequal recognition. Bivariate correlation analysis showed significant correlations among all variables at p < .01, except performance climate and autonomous behavior, perceived motor competence with cooperative learning, punishment of mistakes and unequal recognition, emphasis on effort and improvement with punishment of mistakes and intrateam member rivalry and competition.

**Table 2:** Descriptive analysis and correlations.

		R	M	SD	A	K	❬	1	2	3	4	5	6	7	8	9	10
**1**	**Mastery climate**	1–5	4.05	.59	-.80	.89	.91	-	-.310*	.114*	.457*	.833**	.895**	.783**	-.099*	-.096*	-.427**
**2**	**Performance climate**	1–5	2.85	.77	.10	-.50	.88	-	-	.039	-.422*	-.259**	-.270**	-.253**	.803**	.647**	.915**
**3**	**Perceived motor competence**	1–7	4.13	.92	-.02	.06	.89	-	-	-	.210*	.054	.166**	.116**	.003	.099*	.028
**4**	**Autonomous behavior**	1–7	4.08	1.43	-.18	-.56	.85	-	-	-	-	.336**	.466**	.325**	-.291**	-147**	-466**
**5**	**Cooperative learning**	1–5	4.01	.82	-.85	.16	.87	-	-	-	-	-	.611**	.502**	-107**	-.086*	-337**
**6**	**Emphasis on effort and improvement**	1–5	4.04	.61	-.69	.60	.92	-	-	-	-	-	-	.558**	-.071	-.061	-392**
**7**	**Perceived important role**	1–5	4.10	.72	-.87	.60	.95	-	-	-	-	-	-	-	-.077	-104**	-341**
**8**	**Punishment of mistakes**	1–5	2.95	.88	.07	-.43	.86	-	-	-	-	-	-	-	-	.344**	.554**
**9**	**Intrateam member rivalry and competition**	1–5	3.04	.79	-.25	.13	.84	-	-	-	-	-	-	-	-	-	.494**
**10**	**Unequal recognition**	1–5	2.68	1.06	.15	.943	.86	-	-	-	-	-	-	-	-	-	-

* p < .01; M = Mean; SD = Standard deviation; A = Asymmetry; K = Kurtosis; ❬ = Cronbach’s alpha.

### Cluster analysis to obtain motivational profiles

**Fig 1:**
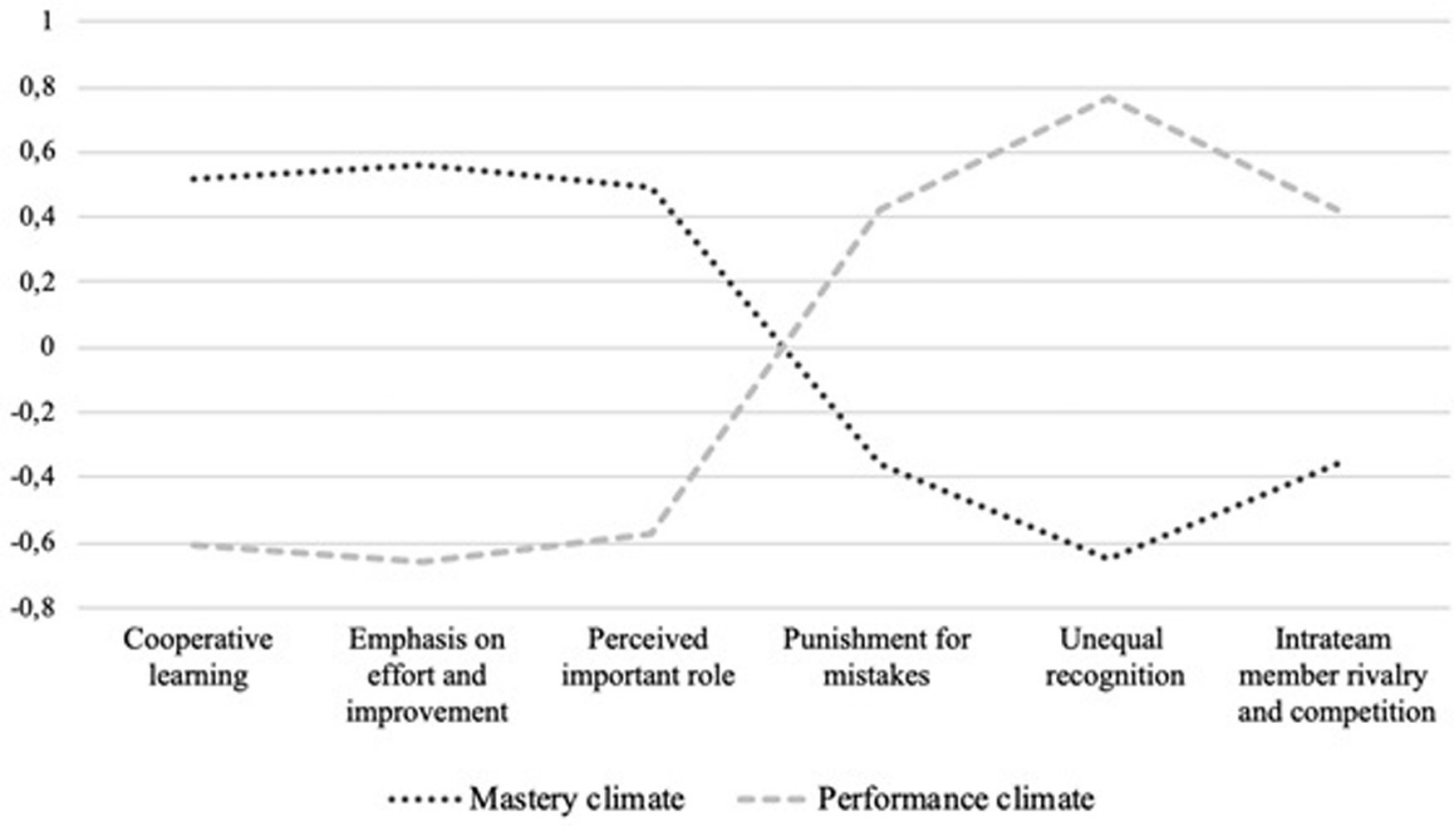
Ward’s hierarchical clustering method to team sports players.

The cluster analysis was conducted including the six subscales of the motivational climate variable, following the phases proposed by Hair et al. [48]. The values of the variables were cohesioned using Z-scores, none of them being higher than 3 and, therefore, no outliers existed in the whole sample. The dendogram obtained suggested the existence of two groups or profiles (see Fig 1). To decide about its adequacy, the number of clustering coefficients was increased by changing from two to three groups. It was concluded that there existed two different groups of players who perceived different motivational climates. Firstly, a ‘‘performance climate” profile (cluster 1), composed of 283 players (46.0%) with the highest scores, sorted by order of score, in unequal recognition (Z = .76) punishment for mistakes (Z = .44), and intrateam rivalry and competition (Z = .42). And secondly, a ‘‘mastery climate” profile (cluster 2), including 332 players (54.0%) with higher scores, sorted by order of score, emphasis on effort and improvement (Z = .56), cooperative learning (Z = .51), and perceived importance of role (Z = .48).

### Differences based on perceived motor competence and autonomous behavior

A MANOVA was conducted to identify the characteristics of each one based on the other variables. Clusters were used as independent variables, and perceived motor competence and autonomous behavior as dependent variables. Box’s test was applied to check the homogeneity of covariance. The results (see Table 3) revealed that the groups did differ in the set of variables (Pillai’s trace = .197; F(75.25); p < .01). Furthermore, follow-up ANOVAs revealed no significant difference in the perceived motor competence but significant differences in autonomous behavior, with mastery climate scoring higher values.

**Table 3:** Multivariate analysis according to cluster based on the scales ASCQ and PCLDS.

	Performance climate (n = 283)			Mastery climate (n = 332)					
	*M*	*SD*	*Z*	*M*	*SD*	*Z*	F	p	⎧
**1 Perceived motor competence**	4.13	.96	.00	4.13	.89	.00	.001	.976	.00
**2 Autonomous behavior**	3.41	1.32	-.46	4.66	1.26	.40	238.73	.000**	.18

Cluster 1: performance climate; Cluster 2: mastery climate; M = mean; SD = standard deviation; Z = standardized mean; ⎧ = partial eta squared;

** p < .01.

Table 4 shows the association of game category, sport experience and training hours. Regarding players’ age and sport experience, mastery climate was positively associated with the junior category (X2 = 22.058; df = 1; p < .01), and players with less than 10 years of sport experience (X2 = 5.082; df = 1; p < .05), while performance climate was associated positively with the senior category and players with more than 10 years of experience. On the other hand, it did not show any differences concerning the training hours, with similar proportions in each profile (X2 = .c610; df = 1; p > .05).

**Table 4:** Profile’s characteristics based on game category, sport experience and training hours.

		Category			Sport experience			Training hours (per week)		
		Junior	Senior	p	- 10 y	+ 10 y	p	- 8 h	+ 8 h	*p*
**Performance climate**	N	83	200	.000**	148	119	.024*	215	68	.435
	%	34.3	53.6		42.4	52.0		45.2	48.9	
**Mastery climate**	N	159	173		201	110		261	71	
	%	65.7	46.4		57.6	48.0		54.8	51.1	

N = frequency; % = percentage;

* p < .05;

** p < .01.

## Discussion

The objectives of the present study were to analyse the motivational profiles that exist in elite sports women who participate in team sports, looking at the perceived motivational climate. Linked to this objective, we also considered seeing if the profiles were related to the idea of a generally autonomous behaviour and with the motor competency. Finally the existing differences according to the level of play were also contrasted, together with the sport experience and the weekly training schedule.

In this sense, the motivational climate generated by the trainer is fundamental to create a positive climate amongst colleagues, which is especially relevant in team sports [49, 50]. In the present study the results suggested the existence of two profiles clearly different in function to that of the perceived motivational climate, in the same way as in previous studies on sports teams [3, 24]. On the other hand, in the contexts of physical education classes, other authors [51, 52] found four profiles. Thus, the analysis of perceived motivational climate profiles can generate different groupings depending on the sample and context being analysed. The groupings in two clusters appear to be the most common in high performance sport.

Looking at the second objective, it was observed that the motivational climate related to mastery was the one with the most benefits to do with improvement in team cohesion. Something which coincides with most of the studies checked [53, 54].

Remember that this motivational climate improves the relationship between the athlete and the trainer [55], increases motivational self-discipline in the athlete [56], boosts the belief in their own ability, and raises the intention of being physically active in the future [57], as well as different over-arching aspects like physical effort [23]. It is worth pointing out that other studies carried out on top level sports-women have demonstrated that females are more highly orientated towards the task in sports such as basketball [31] and football [30].

When looking at the perception of autonomy or perceived autonomous behaviour in athletes, the results of the study demonstrate that the task motivational climate or mastery climate, had significant differences (M = 4.66 vs 3.41) with respect to the profile of ego climate or implementation climate. Various studies have proven these aspects over the years, clearly showing how the involvement towards the task and the release of autonomy of the trainer improves the perception of autonomy of the athletes [11, 20, 58]. However, not all the studies have found this relationship. For example, Morrillo et al. [59], found negative correlations between motivational climate when it involved the task and the perception of autonomy.

For this reason, to continue investigating along these lines is a fundamental aspect if we want to improve the perception of autonomy and the capacity to resolve problems in athletes, which is necessary in certain sports, such as team sports, where interaction between participants takes place.

When it comes to the perception of competence, the results do not show significant differences between both motivational profiles. In previous studies, like the one carried out by Alesi et al. [20], the opposite happened. That is, the three basic psychological needs were satisfied. Furthermore, Morillo et al. [59] found a positive relationship between the climate related to ego and the perception of competence, but not with the rest of variables analysed. This could explain why we find that the involvement of ego does not differ to the involvement of task in this variable studied, as it is possible that in the context of high sports performance the existence of this involvement climate is fundamental in order to face the varied competitive situations [12]. Following these aspects of literature, it can be confirmed that the results are normal, especially due to the type of sample analysed, with the fact that the perception of competence is the least affected, of the three basic psychological needs, by a motivational climate about ego [12]. The last of the objectives tried to analyse the differences between the motivational profiles of the sociodemographic variables who were the object of the study. Firstly, looking at the category of play, the results showed that the youngest players (juniors) were the ones who made the largest contribution to the profile of involvement towards task, which is what also happened in the sports experience. In this way, we agree with previous studies, where the performance climate increases as time passes, in the same way as the sports experience of the athlete, while the mastery climate goes down [12]. Conversely, various authors [29, 60, 61], when working with adolescents, found no significant differences related to the age in this variable. Secondly, when it comes to hours of training, no significant differences were found in the variables analysed. What is more, there are very few studies which have analysed this variable, which have a big influence on the sport output, making our study stand out, as it differs, in part, to that of Manzano-Sánchez et al. [33], where the age and the hours of training were the biggest predictors of the perception of a motivational climate of mastery in both sexes, and finally, in females, the only predictor was the hours of training per week.

On the other hand, the present study had a series of limitations which should be taken into account. On the one hand the sample was accessible and convenient. It would also have been interesting to analyse men in order to see contrasts and the differences between genders. At the same time there were only two categories of play, and it could have been interesting to analyse the perceived motivational climate during the formation process of the player, checking, in this way, if differences exist with younger players.

Finally, it is worth pointing out that the analysed motivational climate was that perceived by the athletes, which it would have been interesting to contrast with the one that the trainers believed they instilled in their training sessions. Future research could reduce these limitations, carrying out studies similar to this one, while analysing different variables, which have been widely proven by scientific literature. Likewise, it would also be interesting to know if the characteristics of the sample could have influenced the results obtained, that is, it would be necessary to contrast the results with similar studies in other samples from other team sports, as well as individual disciplines. On the other hand, it would be interesting to see aspects like the attribution of performance depending on the motivational orientation [31], or include the basic psychological necessity of social relationships, as it has been seen that socialisation plays an important part in the evaluation of the level of performance and the commitment in women’s football [62].

## Conclusions

This study provides relevant and novel information on the motivational climate generated by coaches and perceived by high-performance athletes and its implication in perceived motor competence and autonomous behavior. The bibliographic review carried out shows the scarcity of similar studies published in Spain and more specifically in a sample with these characteristics. It should also be noted that it is difficult to carry out a study of this nature. The results found provide information about the role of the coach as a socializing agent and its importance in improving the perception of motor competence and autonomous behavior of the players during their sports practice.

The mastery motivational climate was the predominant factor in the sample of elite female team players, and the one which showed superior values in the perception of autonomy with respect to the performance climate, with no significant differences found in motivational climate with respect to perceived motor competence. The sports experience of the player has no correlation with the mastery climate, which is predominant in sports players with less than ten years’ experience, or in the junior category. On the other hand, the performance climate was the predominant factor in the senior category, and in those sports players with more than ten years’ experience. While no significant differences in the values relative to hours of training during weekly sessions were found. Therefore, the perception of a mastery climate is the most appropriate to satisfy the basic psychological needs of perception of autonomy and competence. For this reason, the actions of the trainer will be reflected, and will have significant consequences in the perception of support of autonomy and perceived competence, but not in a superior way to the involvement towards the task.

This information will allow coaches to individualize their planning and behavior with their players. The following practical applications can be highlighted to favor the perception of competence and autonomy in female athletes. To encourage players to feel competent or skilled in the sport they play, coaches must, firstly, correctly plan the aims of their programming and training sessions, for which they must know the capabilities of their players and adjust the demands according to their level. Secondly, coaches must provide technical and positive feedback. Thirdly, they must transmit competence in their sport and finally, they must get the players to focus on improving their own task and avoid external pressures that cause them stress. On the other hand, in order to favor the players’ ability to make decisions and be autonomous during training sessions and competition, coaches must take care of the information they offer to their players (before, during -feedback- and after training sessions and competitions), giving the players the possibility to decide, involving the players in certain decisions during training sessions, encouraging the players to express their opinion, and developing self-control and self-direction techniques.

## Supporting information

S1 File (SAV)
